# C-X-C Motif Chemokine 10 Contributes to the Development of Neuropathic Pain by Increasing the Permeability of the Blood–Spinal Cord Barrier

**DOI:** 10.3389/fimmu.2020.00477

**Published:** 2020-03-20

**Authors:** Hao-Ling Li, Yan Huang, Ya-Lan Zhou, Run-Hua Teng, Shu-Zhuan Zhou, Jia-Piao Lin, Yan Yang, Sheng-Mei Zhu, Hua Xu, Yong-Xing Yao

**Affiliations:** ^1^Department of Anesthesia, First Affiliated Hospital, Zhejiang University School of Medicine, Hangzhou, China; ^2^Department of Anesthesia, Changhai Hospital, Second Military Medical University, Shanghai, China; ^3^Department of Anesthesia, The Central Hospital of Lishui City, Lishui, China; ^4^Centre for Neuroscience, Zhejiang University School of Medicine, Hangzhou, China; ^5^Department of Anesthesia, Yueyang Hospital of Integrated Traditional Chinese and Western Medicine, Shanghai University of Traditional Chinese Medicine, Shanghai, China

**Keywords:** neuropathic pain, chronic constriction injury, blood–spinal cord barrier, neuroinflammation, behavior, C-X-C motif chemokine 10

## Abstract

Neuropathic pain is among the most debilitating forms of chronic pain. Studies have suggested that chronic pain pathogenesis involves neuroimmune interactions and blood–spinal cord barrier (BSCB) disruption. However, the underlying mechanisms are poorly understood. We modeled neuropathic pain in rats by inducing chronic constriction injury (CCI) of the sciatic nerve and analyzed the effects on C-X-C motif chemokine 10 (CXCL10)/CXCR3 activation, BSCB permeability, and immune cell migration from the circulation into the spinal cord. We detected CXCR3 expression in spinal neurons and observed that CCI induced CXCL10/CXCR3 activation, BSCB disruption, and mechanical hyperalgesia. CCI-induced BSCB disruption enabled circulating T cells to migrate into the spinal parenchyma. Intrathecal administration of an anti-CXCL10 antibody not only attenuated CCI-induced hyperalgesia, but also reduced BSCB permeability, suggesting that CXCL10 acts as a key regulator of BSCB integrity. Moreover, T cell migration may play a critical role in the neuroimmune interactions involved in the pathogenesis of CCI-induced neuropathic pain. Our results highlight CXCL10 as a new potential drug target for the treatment of nerve injury–induced neuropathic pain.

## Introduction

Neuropathic pain is caused by primary lesions or dysfunction in the nervous system, and it is among the most debilitating forms of chronic pain (1). Its etiology is poorly understood, and this hinders the development of therapeutic and preventative strategies (2–4). One thing that is clear is that peripheral nerve injury leads to neuropathic pain by triggering radical changes that affect multiple components of the pain signaling pathway (5, 6).

Over the past decade, inflammatory responses after nerve injury have become an important topic in neuropathic pain research, and recent evidence suggests that neuroimmune interactions are involved in the pathogenesis of chronic pain states (7–9). Accumulating evidence indicates that multiple proinflammatory mediators are released from injured nerve fibers and adjacent immune cells after nerve injury and that these mediators in turn promote central sensitization and behavioral hyperalgesia (5, 10). Furthermore, animal studies have shown that peripheral nerve injury induces circulating immune cells to enter the spinal cord parenchyma, a phenomenon that may contribute to pain-related behaviors during the development of neuropathic pain (11–13).

Because the blood–spinal cord barrier (BSCB) is the main structure regulating interactions between the immune system and the central nervous system (CNS), it is reasonable to speculate that BSCB dysfunction may play a critical role in the migration of circulating immune cells into the spinal cord (14). However, little is known about the functional states of the BSCB in the context of peripheral nerve injury–induced neuropathic pain. Researchers do not know how, or even whether, a remote injury can affect BSCB integrity. The potential consequences of compromised BSCB integrity in terms of spinal cord homeostasis and the development of pathological pain are also unclear.

This matter may be illuminated through research into chemokines, a family of small cytokines (i.e., signaling proteins) that are upregulated by primary proinflammatory mediators and tumor necrosis factors (15, 16). In the CNS, chemokines regulate myriad functions including neuronal development, synaptic transmission, and neuroinflammation (17–20). Recent studies have shown that the C-X-C motif chemokine receptor 3 (CXCR3) and its ligand C-X-C motif chemokine 10 (CXCL10) are involved in the pathophysiology of allergic itches and neuropathic pain (21–23). However, the mechanism by which CXCL10/CXCR3 signaling mediates neuropathic pain remains poorly understood.

Past studies have reported that CXCL10 promotes the entry of peripheral immune cells into the spinal cord (24, 25). On the other hand, some studies and our recent report have shown that T cell infiltration of the dorsal horn may contribute to the onset of neuropathic and inflammatory hyperalgesia (11, 12, 26, 27). However, it is unknown whether these processes contribute to hyperalgesia following peripheral nerve injury.

In this study, we examined the integrity of the BSCB and the migration of circulating immune cells into the spinal cord after chronic constriction injury (CCI) of the sciatic nerve, which induces neuropathic pain. We also examined the activation of the CXCL10/CXCR3 signaling pathway after CCI. We aimed to elucidate the pathophysiology underlying nerve injury-induced neuropathic pain and to identify potential drug targets for the treatment of neuropathic pain.

## Materials and Methods

### Ethics

All animal experiments were conducted in accordance with the ARRIVE guidelines (28) and all relevant Chinese laws. The experimental protocol was approved by the Research Ethics Committee of the First Affiliated Hospital at Zhejiang University. All measures were taken to minimize the animals' suffering and to reduce the number of animals used.

### Animals

Adult male Sprague–Dawley rats (87 rats in total, 8 weeks at arrival) weighing 200–300 g were obtained from the Animal Center of the Chinese Academy of Sciences. They were housed in groups (4 rats/cage) in a temperature-controlled room (22 ± 2°C) with a 12-/12-h light/dark cycle and *ad libitum* access to food and water.

### CCI Induction

The rats were randomly divided into the sham surgery and CCI groups (*n* = 5–6 per group for the behavioral test; *n* = 3–4 for the others). After the baseline was determined, the rats underwent the corresponding procedures on experimental day 0. CCI was surgically induced as described in our previous publication (29) and another study (30). In brief, the rats were anesthetized with intraperitoneal pentobarbital injections (60 mg/kg), and the left sciatic nerve was exposed and isolated. Three ligations were placed around the nerve with 4–0 chromic gut sutures (Pudong Jinghuan Co. Ltd., Shanghai, China). A hindpaw twitch indicated successful nerve constriction. The muscles and skin overlying the sciatic nerve were then closed with sutures. The sham surgery was identical except for the omission of sciatic nerve ligation. All animals received hypodermic penicillin injections (0.5 mL/rat; 96 mg/mL) to reduce the risk of infection. To reduce variability, all surgeries were performed by a single proficient investigator.

### Immunohistochemistry

The rats were anesthetized with an intraperitoneal injection of pentobarbital (60 mg/kg) and perfused with normal saline (NS), followed by 4% ice-cold paraformaldehyde in phosphate buffer. The lumbar 4–5 segments were removed, post-fixed, and dehydrated in 30% sucrose at 4°C. Next, 30-μm free-floating transverse cutting was performed using a freezing microtome at −20°C. After blocking with 10% goat serum for 2 h at room temperature to reduce non-specific binding, the sections were incubated for 48–72 h with the following primary antibodies: mouse anti-CXCR3 (1:100 dilution, Santa Cruz Biotechnology, Dallas, TX, USA), rabbit anti-Iba1 (1:400; Abcam, Cambridge, UK), rabbit anti-GFAP (1:500; Proteintech, Rosemont, IL, USA), and rabbit anti-NeuN (1:400, Cell Signaling Technology, Danvers, MA, USA). Subsequently, the sections were incubated with an appropriate secondary antibody (FITC-conjugated goat anti-rabbit or Cy3-conjugated goat anti-mouse, both 1:200 dilution; Beyotime, Shanghai, China) for 2 h at room temperature in the dark. Fluorescence signals were observed using a fluorescence microscope with appropriate filters.

### Western Blotting

After the intraperitoneal injection of an overdose of pentobarbital, the spinal dorsal horn segments (lumbar 4–5) were dissected rapidly and stored in liquid nitrogen. Frozen samples were homogenized in lysis buffer containing PMSF (Beyotime). After centrifugation at 10,600 rpm and 4°C for 15 min, the supernatants were collected as protein samples. Sample aliquots containing equal amounts of protein were separated via SDS-PAGE and transferred onto polyvinylidene difluoride membranes. The membranes were blocked in 5% non-fat milk for 1 h at room temperature, and incubated overnight at 4°C with rabbit anti-CXCR3 (1:500 dilution; Abcam), rabbit anti-CXCL10 (1:1,000; GeneTex, Irvine, CA, USA), or mouse anti-GAPDH (1:10,000; Proteintech). The membranes were then washed with TBST buffer and incubated with an appropriate secondary antibody (horseradish peroxidase-conjugated goat anti-mouse or goat anti-rabbit, 1:2,000; Beyotime) for 2 h at room temperature. After extensive washing, the densities of labeled protein bands on the blots were detected using an enhanced chemiluminescence reagent (Thermo Fisher, Waltham, MA, USA) and captured using a ChemiDoc MP System (Bio-Rad, Hercules, CA, USA).

### Anti-CXCL10 Antibody Administration and Timeline of the Experiments

To investigate the role of CXCL10/CXCR3 signaling in CCI-induced neuropathic pain, rats were randomly divided into the sham surgery (*n* = 5–6) and CCI groups. Subgroups of rats that underwent CCI were selected to receive intrathecal antibody or saline injections (*n* = 5). Intrathecal administration was performed by lumbar puncture, as described in a previous study (31). The rats were anesthetized with isoflurane (0.3 mL/rat, Baxter International Inc.; Shanghai, China) in a home-made anesthesia box and placed on a plexiglass tube to broaden the intervertebral spaces. A 20-μL volume of normal saline or a solution containing an anti-CXCL10 antibody (200 ng/rat, Proteintech) was injected into the subarachnoid space between the L5 and L6 vertebrae with a 30-gauge needle. An instantaneous and rapid tail-flick indicated a successful puncture.

To determine the effect of CXCL10/CXCR3 signaling on the development of hyperalgesia, the first injection of anti-CXCL10 antibody was administered on experimental day 1 after CCI. Daily follow-up injections were performed until day 14 (for the behavioral experiment), unless the rats were sacrificed earlier for a BSCB permeability evaluation or flow cytometry assay. To determine the effect on established hyperalgesia, the injections were administered on experimental days 5–7 after CCI. Rats that received normal saline injections are herein referred to as the CCI + NS group, while those that received anti-CXCL10 antibody injections are herein referred to as the CCI + anti-CXCL10 antibody group.

### Von Frey Test for Hypersensitivity to Mechanical Stimulation

Hyperalgesia was assessed based on paw withdrawal responses to a calibrated series of von Frey filaments (Stoelting; Wood Dale, IL, USA) as described in one of our earlier publications (32). In brief, the rats were individually placed in a chamber (20 cm × 10 cm × 20 cm) in which the floor was a customized platform consisting of a grid of iron wires with 10-mm spacings between wires. The rats were allowed to acclimate to the chamber for ≥30 min before the experiment began. A series of von Frey filaments with ascending buckling forces were applied to the midplantar surface of the hindpaw ipsilateral to the site of the CCI or sham surgery (herein referred to as the ipsilateral hindpaw) and the hindpaw contralateral to the surgical site (herein referred to as the contralateral hindpaw). Each von Frey filament was held for 2 s, and the interval between filament applications was 15 s. A brisk withdrawal or flinching of the hindpaw upon filament application was regarded as a positive response, and the filament applications continued until a filament produced positive responses in at least three out of five consecutive applications. The paw withdrawal threshold (PWT) was defined as the buckling force (in grams) of that particular filament.

PWT testing was performed by an investigator who was blinded to the rats' group assignments. Daily PWT testing began on experimental day 0 (baseline) and continued until day 14 after CCI or sham surgery.

### BSCB Permeability Evaluations

BSCB permeability was assessed with the micromolecular tracer dye sodium fluorescein (NaFlu; molecular weight, 376 g/mol; Sigma-Aldrich; St. Louis, MO, USA) according to a modified version of a published procedure (33). In brief, subgroups of rats that underwent sham or CCI surgeries were selected to receive intravenous injections of a 10% NaFlu solution (2 μL per gram of bodyweight) on experimental day 3. After an intraperitoneal injection of pentobarbital (60 mg/kg), NaFlu was allowed to circulate for 30 min, and the rats' bodies were then intracardially perfused with cold saline to remove intravascular NaFlu. The L4 and L5 spinal cord segments were removed and used for subsequent analyses aimed at quantifying the amount of NaFlu extravasated from circulation.

After being weighed, the spinal cord samples were homogenized in 1 mL of phosphate-buffered saline (PBS), and a volume of 60% trichloroacetic acid equal to that of the resulting solution was added to precipitate proteins. After being vortexed for 2 min, the samples were cooled for 30 min and centrifuged at 14,000 × g for 10 min. The NaFlu concentration in the supernatant was measured with a spectrophotofluorometer (excitation wavelength, 440 nm; emission wavelength, 525 nm). A calibration curve was created by assaying solutions with controlled NaFlu concentrations under identical assay conditions. All experimental measurements were within the detection range established with the calibration curve, which had an *R*^2^-value of 0.85–0.90. NaFlu levels were calculated as micrograms per gram of spinal cord tissue.

### Flow Cytometry

To assess T cell entry into the spinal cord after CCI, flow cytometry was used to measure CD3-positivity levels in mononuclear cells extracted from the dorsal horn, as CD3 is a well-known T cell marker (34). On day 3 of CCI, after an overdose intraperitoneal injection of urethane (2 g/kg), the rats' lumbar spinal cord segments were harvested. The dorsal horn tissues ipsilateral to the site of CCI or sham surgery were isolated, placed in 0.01-M PBS, and homogenized into single-cell suspensions with a cell strainer. Homogenates were washed with 0.01-M PBS, suspended in a 30%/70% discontinuous-gradient Percoll solution (Sigma-Aldrich), and centrifuged at 390 × g for 30 min.

Mononuclear cells were collected, washed with 0.01-M PBS, and resuspended in a fluorescence-activated cell sorting buffer solution for 30 min at 4°C. The cells were labeled with fluorescein isothiocyanate–conjugated mouse anti-CD3 antibodies (1:100 dilution; eBioscience, San Diego, CA, USA) for 20 min at room temperature, and ≥10,000-cell samples were analyzed with the FACSCalibur platform running with CellQuest software (Becton Dickinson; Franklin Lakes, NJ, USA) to determine the percentage of mononuclear cells that were CD3-positive.

### Statistical Analyses

Statistical analyses were performed with Prism 5.0 software (GraphPad Software; La Jolla, CA, USA). Data were expressed as means ± standard errors. Behavioral data were analyzed with two-way repeated-measures analysis of variance (ANOVA) followed by Bonferroni *post-hoc* testing. BSCB permeability evaluation and flow cytometry data were analyzed with the independent *t-*test. Mann–Whitney *U-*test was used if equal-variance assumptions were not made. Statistical significance was defined as *p* < 0.05.

## Results

### Colocalization of CXCR3 With Spinal Neurons

**Figure 1:**
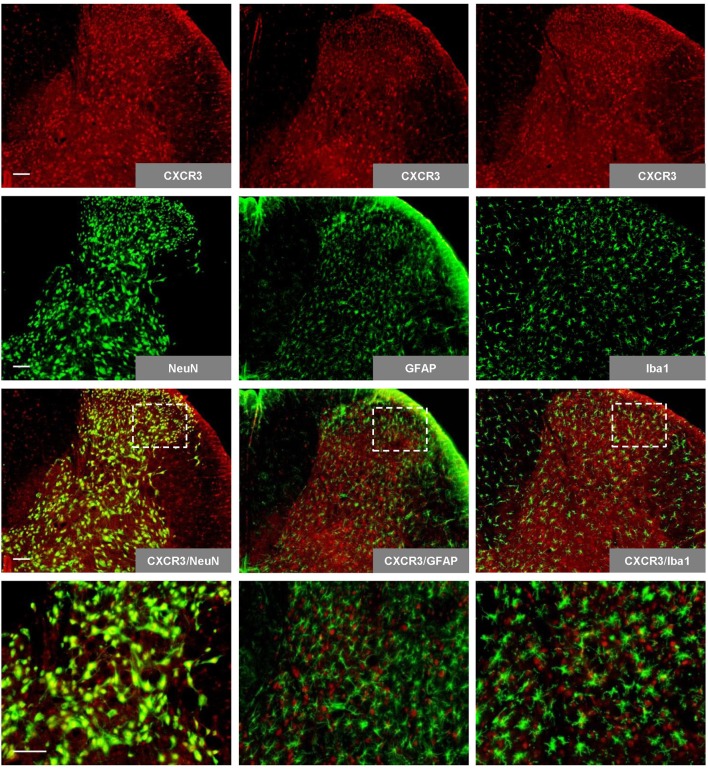
Expression and colocalization of CXCR3 with spinal neuron markers. Spinal CXCR3 is expressed abundantly in the spinal dorsal horn, where it colocalizes with NeuN (neuron marker), but not with GFAP (astrocyte marker) or Iba1 (microglia marker). The last row presents enlargements of the areas in white frames. Scale bar = 100 μm. NeuN, neuronal nuclear antigen; GFAP, glial fibrillary acidic protein; Iba1, ionized calcium binding adapter molecule.

We initially performed immunohistochemistry for specific cell markers to determine the profile of CXCR3 expression in the spinal cord. The results revealed that CXCR3 is expressed abundantly in the spinal cord, where it colocalized with NeuN (neuron marker), but not with GFAP (astrocyte marker) or Iba1 (microglia marker; Figure 1).

### Effects of CCI on PWTs and BSCB Permeability

**Figure 2:**
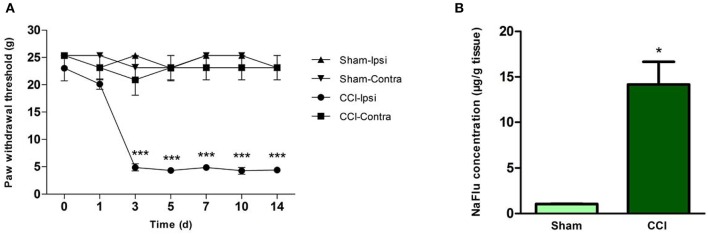
CCI resulted in behavioral hyperalgesia and BSCB disruption. **(A)** PWTs of hindpaws ipsilateral and contralateral to surgical sites in the sham surgery group and CCI group rats on various experimental days (***p < 0.001; ANOVA, *n* = 6). **(B)** Lumbar spinal cord NaFlu concentrations in the sham surgery group and CCI group rats on experimental day 3 (*p < 0.05; Mann–Whitney *U-*test, *n* = 4). The data are shown as means ± standard errors. ANOVA, analysis of variance; BSCB, blood-spinal cord barrier; Contra, contralateral; CCI, chronic constriction injury; Ipsi, ipsilateral; NaFlu, sodium fluorescein; PWT, paw withdrawal threshold.

Relative to the sham surgery group rats, the CCI group rats had decreased ipsilateral hindpaw PWTs on experimental days 3, 5, 7, 10, and 14 (*p* < 0.001; *n* = 6, ANOVA, Figure 2A), indicating CCI-induced mechanical hyperalgesia. No significant between-group differences were observed for contralateral hindpaw PWTs (Figure 2A). The CCI group rats also had dramatically elevated lumbar spinal cord NaFlu concentrations on experimental day 3 (*p* = 0.029; *n* = 6, Mann–Whitney *U-*test, Figure 2B), which suggests that CCI increased BSCB permeability.

### Effects of CCI on CXCL10/CXCR3 Signaling Activation

**Figure 3:**
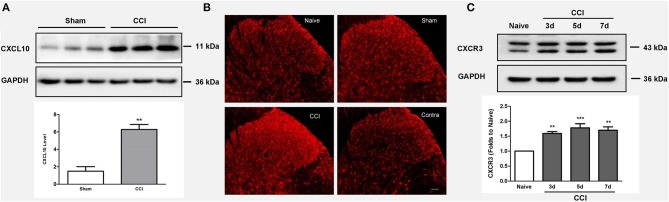
CCI induces CXCL10/CXCR3 signaling activation. **(A)** Upregulated CXCL10 expression is observed in the ipsilateral spinal cord after CCI injury (**p < 0.01; independent *t*-test, *n* = 3). **(B)** Immunohistochemistry reveals the CCI-mediated induction of CXCR3 activation. **(C)** The spinal CXCR3 protein level increased during days 3–7 after CCI as shown by western blotting (**p < 0.01; ***p < 0.001; ANOVA, *n* = 3). The data are presented as means ± standard errors. GAPDH was used as a loading control. ANOVA, analysis of variance; CCI, chronic constriction injury; CXCL10, C-X-C motif chemokine 10; Contra, contralateral to CCI.

Compared to the sham surgery group, we observed increased CXCL10 expression in the ipsilateral spinal cord after CCI injury (*p* = 0.003; *n* = 3, independent *t-*test, Figure 3A). The immunohistochemical results revealed that CCI induced CXCR3 activation, as shown in Figure 3B. This was further confirmed by western blotting, which demonstrated that the CXCR3 protein level was increased from 3 to 7 days after CCI (*p* < 0.01; *n* = 3, ANOVA, Figure 3C).

### Effects of CXCL10/CXCR3 Signaling Blockade on CCI-Induced Hyperalgesia

**Figure 4:**
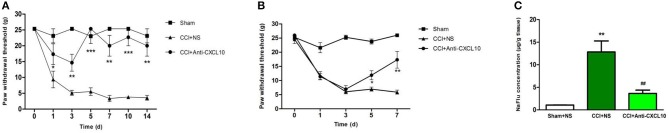
Neutralizing CXCL10 alleviated hyperalgesia and reduced CCI-induced BSCB disruption. **(A,B)** Hindpaw PWTs in CCI + anti-CXCL10 antibodies group and CCI + saline group rats on various experimental days (*p < 0.05, **p < 0.01, ***p < 0.001; ANOVA, *n* = 5–6). **(C)** Lumbar spinal cord NaFlu concentrations in the CCI + anti-CXCL10 antibodies group, CCI + saline group, and sham surgery group rats on experimental day 3 (*p < 0.01, CCI + NS group vs. sham surgery group; Mann–Whitney *U-*test, *n* = 4–6; ^##^*p* < 0.01, CCI + anti-CXCL10 antibodies group vs. CCI + NS group; independent *t-*test, *n* = 6). The data are shown as means ± standard errors. ANOVA, analysis of variance; BSCB, blood-spinal cord barrier; CCI, chronic constriction injury; CXCL10, C-X-C motif chemokine 10; NaFlu, sodium fluorescein; PWT, paw withdrawal threshold; NS, normal saline.

Relative to the rats treated with CCI + saline group, rats in the CCI + anti-CXCL10 antibody group exhibited a marked increase in PWTs from experimental day 1 to day 14 or from day 5 to 7 (*p* < 0.05, *n* = 5, ANOVA, Figures 4A,B). This indicates that the anti-CXCL10 antibody attenuated CCI-induced mechanical hyperalgesia in both the developmental and established stages. On the other hand, anti-CXCL10 antibody dramatically reduced NaFlu concentrations in the lumbar spinal cord on experimental day 3 (*p* = 0.0049; *n* = 6, independent *t-*test, Figure 4C). These results suggest that the CXCL10/CXCR3 signaling pathway is involved in the pathophysiology of CCI-induced hypernociception and increased BSCB permeability.

### T Cell Entry Into the Spinal Cord After CCI

**Figure 5:**
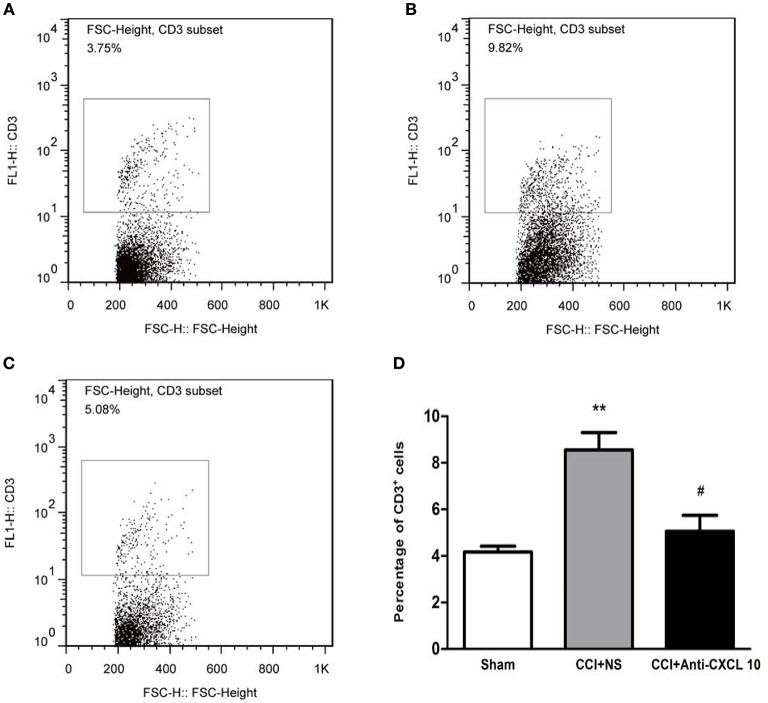
Neutralizing CXCL10 reduced CCI-induced T cell infiltration of the dorsal horn. **(A,B)** CD3-positive T cell levels as percentages of CCI-ipsilateral dorsal horn mononuclear cell populations in the sham surgery group and CCI + NS group rats (**p < 0.01; independent *t-*test, *n* = 3–4). **(B,C)** CD3-positive T cell levels as percentages of CCI-ipsilateral dorsal horn mononuclear cell populations in CCI + NS group and CCI + anti-CXCL10 antibodies group rats (^#^*p* < 0.05; independent *t-*test, *n* = 3). **(D)** Bar chart indicating the statistical analysis. The data are shown as means ± standard errors. CCI, chronic constriction injury; CXCL10, C-X-C motif chemokine 10; NS, normal saline.

The percentage of ipsilateral dorsal horn mononuclear cells that were CD3-positive was more than two times higher in the CCI group rats than in the sham surgery group rats (*p* < 0.0014; *n* = 3–4, independent *t-*test, Figures 5A,B) and was lower in the CCI + anti-CXCL10 antibodies group rats than in the CCI + NS group rats (*p* < 0.0264; *n* = 3, independent *t-*test, Figures 5B,C). These results suggest that CCI promotes T cell entry into the spinal cord, and that blocking the CXCL10/CXCR3 signaling pathway counteracts this effect, which provides further evidence for the CXCL10/CXCR3 signaling pathway being involved in the pathophysiology of CCI-induced BSCB disruption (Figure 5D).

## Discussion

In this study, we investigated the putative link between CXCL10/CXCR3 signaling-mediated BSCB disruption and neuropathic pain. As in our previous studies, CCI group rats exhibited robust post-operative behavioral hypersensitivity to mechanical stimuli, and this hypersensitivity persisted throughout the experimental period (29, 32). We also determined that CCI induced CXCL10/CXCR3 signaling, increased BSCB permeability, and promoted T cell migration into the spinal dorsal horn. Moreover, intrathecal administration of anti-CXCL10 antibodies attenuated the rats' behavioral hyperalgesia and reduced the CCI-induced increases in BSCB permeability and T cell infiltration into the dorsal horn. To the best of our knowledge, it is the first study to report that blocking CXCL10/CXCR3 signaling attenuates the increases in BSCB permeability and T cell infiltration of the spinal cord induced by peripheral nerve injury.

Researchers have attempted to unravel the mechanisms underlying CCI-induced inflammatory reactions in the spinal cord, and have made considerable progress (35, 36). Myriad inflammatory mediators in the spinal cord may contribute to the development of neuropathic pain, with interleukin-6, tumor necrosis factor alpha, and C-X-C motif chemokines being possible examples (5, 37, 38). Among the chemokines, CXCL10 has been identified as a potentially important trigger (39, 40), and our results provide further evidence for its importance. Previous studies have indicated that increased BSCB permeability is a prerequisite for immune cell infiltration of the spinal cord during the development of neuropathic pain (41), and we found that blocking CXCL10/CXCR3 signaling with anti-CXCL10 antibodies reduced the BSCB's permeability to NaFlu, which suggests that CXCL10/CXCR3 signaling plays a critical role in CCI-induced BSCB dysfunction.

The chemokines CXCL9, CXCL10, and CXCL11 compose a subfamily of chemokines that bind to CXCR3 and have various roles in nociceptive signaling. Past investigations have suggested that CXCL10/CXCR3 signaling contributes to the pathophysiology of neuropathic pain, although spinal CXCL9 and CXCL11 levels do not seem to have important roles in the development of chronic pain (42, 43). Other reports have shown that T cell infiltration of the dorsal horn may contribute to the onset of neuropathic and inflammatory hyperalgesia (10, 12, 39). Our present findings further this line of research by elucidating the potential mechanistic role of CXCL10/CXCR3 signaling in the development of neuropathic pain following peripheral nerve injury. Our observation that blocking CXCL10/CXCR3 signaling reduced CCI-induced T cell migration into the spinal cord is consistent with past reports suggesting that CXCL10/CXCR3 signaling plays a role in the migration of T cells from the periphery into the CNS (44).

Within the spinal cord, neurons, and glia can secrete CXCL10, which in turn promotes the entry of circulating immune cells into the spinal cord (45–48). Studies have shown that type 1 T helper cells secrete interferon gamma, and elevated spinal cord interferon gamma levels can induce CXCL10 secretion. CXCL10 in turn increases BSCB permeability and promotes the migration of T cells into the spinal cord (44, 49). This creates a positive feedback system that favors ever-increasing migration of activated T cells into the spinal cord. This implies that blocking the contribution of CXCL10/CXCR3 signaling to increased BSCB permeability, as we did by administering anti-CXCL10 antibodies to the CCI group rats, may disrupt this positive feedback loop.

In conclusion, our study suggests that CXCL10/CXCR3 signaling triggers a positive feedback loop involving BSCB permeabilization and T lymphocyte infiltration of the spinal cord. Intrathecal administration of anti-CXCL10 antibodies prevents the development of CCI-induced neuropathic pain. Our findings highlight the CXCL10/CXCR3 signaling pathway as a new potential target for drugs designed to treat chronic pain.

## Data Availability Statement

The datasets generated for this study are available on request to the corresponding author.

## Ethics Statement

The animal study was reviewed and approved by Research Ethics Committee of the First Affiliated Hospital at Zhejiang University.

## Author Contributions

H-LL, Y-XY, and HX contributed to the conception and design of the study. H-LL and YH performed the behavioral analyses. Y-LZ and S-ZZ performed the flow cytometry experiments. R-HT and J-PL performed the histology experiments. Y-XY and YY performed the statistical analyses. H-LL wrote the first draft of the manuscript. S-MZ wrote sections of the manuscript. All authors contributed to manuscript revisions and read and approved the submitted version.

### Conflict of Interest

The authors declare that the research was conducted in the absence of any commercial or financial relationships that could be construed as a potential conflict of interest.
